# Alterations in Cortical Sensorimotor Connectivity following Complete Cervical Spinal Cord Injury: A Prospective Resting-State fMRI Study

**DOI:** 10.1371/journal.pone.0150351

**Published:** 2016-03-08

**Authors:** Akinwunmi Oni-Orisan, Mayank Kaushal, Wenjun Li, Jack Leschke, B. Douglas Ward, Aditya Vedantam, Benjamin Kalinosky, Matthew D. Budde, Brian D. Schmit, Shi-Jiang Li, Vaishnavi Muqeet, Shekar N. Kurpad

**Affiliations:** 1 Department of Neurosurgery, Medical College of Wisconsin, Milwaukee, Wisconsin, United States of America; 2 Department of Biomedical Engineering, Marquette University, Milwaukee, Wisconsin, United States of America; 3 Department of Biophysics, Medical College of Wisconsin, Milwaukee, Wisconsin, United States of America; 4 Department of Neurology, Medical College of Wisconsin, Milwaukee, Wisconsin, United States of America; 5 Department of Neurosurgery, Baylor College of Medicine, Houston, Texas, United States of America; 6 Department of Physical Medicine and Rehabilitation, Clement J. Zablocki Veterans Affairs Medical Center, Milwaukee, Wisconsin, United States of America; University of Toronto, CANADA

## Abstract

Functional magnetic resonance imaging (fMRI) studies have demonstrated alterations during task-induced brain activation in spinal cord injury (SCI) patients. The interruption to structural integrity of the spinal cord and the resultant disrupted flow of bidirectional communication between the brain and the spinal cord might contribute to the observed dynamic reorganization (neural plasticity). However, the effect of SCI on brain resting-state connectivity patterns remains unclear. We undertook a prospective resting-state fMRI (rs-fMRI) study to explore changes to cortical activation patterns following SCI. With institutional review board approval, rs-fMRI data was obtained in eleven patients with complete cervical SCI (>2 years post injury) and nine age-matched controls. The data was processed using the Analysis of Functional Neuroimages software. Region of interest (ROI) based analysis was performed to study changes in the sensorimotor network using pre- and post-central gyri as seed regions. Two-sampled t-test was carried out to check for significant differences between the two groups. SCI patients showed decreased functional connectivity in motor and sensory cortical regions when compared to controls. The decrease was noted in ipsilateral, contralateral, and interhemispheric regions for left and right precentral ROIs. Additionally, the left postcentral ROI demonstrated increased connectivity with the thalamus bilaterally in SCI patients. Our results suggest that cortical activation patterns in the sensorimotor network undergo dynamic reorganization following SCI. The presence of these changes in chronic spinal cord injury patients is suggestive of the inherent neural plasticity within the central nervous system.

## Introduction

Spinal cord injury (SCI) is a serious problem in the United States and worldwide. The global incidence is 40–80 cases per million population with up to 90% of the cases caused by a traumatic event. The estimates for the annual incidence and the total number of people living with SCI in US are approximately 12,500 and 276,000 respectively. The most common cause for the traumatic episode is motor vehicle collisions, implicated as the cause in over a third of the reported cases. Lifetime costs associated with SCI based on 2013 calculations range from 1.1 million– 4.7 million USD depending on factors like patient age, time since injury, level and severity of injury [1].

The spinal cord as part of the central nervous system (CNS) connects the brain and the peripheral nervous system (PNS). SCI interrupts this relationship by damaging sensory and motor nerve bundles functioning as conduits for afferent and efferent impulses respectively. Dysfunction ensues below the spinal cord lesion following the neural disconnect which results in loss of functioning efferent response and generation of appropriate afferent feedback. The intact rostral cortical structures also suffer from loss of afferent feedback and consequently generate impaired efferent response [2–5]. The distortion to input/output balance of neural impulses is transmitted throughout the neuraxis with subsequent structural and functional implications [6–12].

A number of task-induced fMRI studies have demonstrated rearrangement of cortical activation patterns in the form of spatial shifts of activation maps towards secondary brain areas in SCI patients [6, 7, 13–18]. However, task-based fMRI studies lack standardized protocols for application of motor tasks across different SCI injury grades [19]. Resting-state fMRI (rs-fMRI) is unaffected by differences in task protocols as it detects spontaneous low-frequency fluctuations in the blood oxygenation level-dependent (BOLD) contrast signal in the absence of a voluntary activity i.e when the patient is at rest [20]. The demonstration of altered resting-state functional cortical circuitry in conditions like Alzheimer's disease [21, 22], multiple sclerosis [23], amyotrophic lateral sclerosis [24] and stroke [25, 26], further points towards the utility of using rs-fMRI for the functional mapping of the cortical regions in SCI patients.

Animal studies on resting-state connectivity in SCI have demonstrated changes between different regions of sensorimotor cortex [27, 28]. However, how these alterations in activation patterns of the sensorimotor network affect the overall resting state connectivity in SCI patients is not yet understood In humans, studies looking at changes in cortex have been focused on more acute timeframes and have studied multiple spinal cord injury grades [29–31]. To our knowledge, no one has studied the application of rs-fMRI in chronic SCI subset exclusively, with focus on cervical SCI cases. Therefore, we propose a prospective seed-based rs-fMRI study to investigate how the cortical sensorimotor networks are reorganized through neuronal plasticity after complete SCI.

## Methods

### Subjects

Eleven subjects with chronic cervical SCI having injury duration of more than 2 years with no history of associated traumatic brain injury (TBI) and nine age and sex matched, motor and sensory intact, healthy volunteers took part in the study. SCI subjects were recruited at the Froedtert Hospital, Milwaukee, Wisconsin. All participants were scanned at the Center for Imaging Research, Medical College of Wisconsin after signing written consent forms. All procedures were approved by the Institutional Review Boards of the Medical College of Wisconsin and the VA health system. Table 1 provides demographic details about the study participants.

**Table 1:** Demographic and clinical characteristics of study participants.

Characteristics	SCI Patients	Controls
Participants	11	9
Mean age (years)	41.3 ± 15.3	41.6 ± 20.0
Gender (male: female)	11: 0	9: 0
Mean disease duration (years)	11.8 ± 9.1	-
Level of injury (# of participants)	C4(1), C5(1), C6(4), C7(5)	Neurologic intact
Mechanism (# of participants)	MVA(4), Diving(3), Machine(1), Fall(1), MCC(2)	-

SCI: spinal cord injury; MVA: motor vehicle accident; MCC: motorcycle crash.

Unless otherwise indicated, data are presented as mean ± standard deviation.

SCI subjects with American Spinal Injury Association (ASIA) Impairment Scale A (AIS A), corresponding to complete spinal cord injury were selected from a chart review. Inclusion criteria for spinal cord injury patients included: age 18–75 years old, cervical spinal cord injury level and greater than 24 months from time of injury to participation in study. Exclusion criteria included presence of associated traumatic brain injury, decreased cognition and inability to comprehend commands, active bladder or other infection, severe contractures, cardiac arrhythmias on pacemakers or other implanted materials, history of gunshot wounds, eye injuries or any implanted materials not on the approved list for MR compatibility, subjects with metal plates implanted in the head, deformities of the skull or seizure disorders, and inability to consent for procedures. Table 2 summarizes the inclusion and exclusion criteria used for the study.

**Table 2:** Recruitment Criteria.

**Inclusion**
Age 18–75 years
Cervical spinal cord injury
ASIA impairment grade A
Disease duration greater than 24 months
**Exclusion**
Associated TBI, Seizure disorder
Decreased Cognition, Inability to consent
Active Infection
Severe contractures
Cardiac arrhythmias with pacemaker
Hx of gun shot wound
Hx of non MR approved implanted materials

TBI: traumatic brain injury; Hx: history; MR: magnetic resonance.

### MR Imaging and Functional MR Imaging

All SCI subjects were scanned using a whole-body 3.0 T Signa GE scanner (Waukesha, Wisconsin) with a multi channel head and neck coil. For imaging, participants were positioned supine on the gantry of the scanner with the head in a midline location in a purpose built multi-channel head coil and stabilized by clamps to reduce motion related artifact during scanning. During the resting-state acquisitions the study participants were instructed to close their eyes, relax, and stay awake.

High-resolution anatomical images of the brain were obtained using T1-weighted spoiled gradient-recalled (SPGR) pulse sequence with TR = 8.2 ms, TE = 3.2 ms, FOV = 24 cm^2^, image matix = 256 x 192, NEX = 1, slice thickness = 1 mm with no gaps and one excitation-per-phase encoding step for a scan time of 8.5 min. Next, functional imaging was collected, using gradient-echo echo-planar imaging (EPI) pulse sequence with TR = 2000 ms, TE = 25 ms, FOV = 24 cm^2^, image matrix = 64 x 64, bandwidth = 250 kz, slice thickness = 3.5 mm with no gaps, image orientation = sagittal and repetitions (time points) = 300 for a scan time of 8 min. A total of 8640 images were obtained with voxel resolution of 3.75 x 3.75 x 4 mm^3^.

### Preprocessing of scanner data

**Fig 1:**
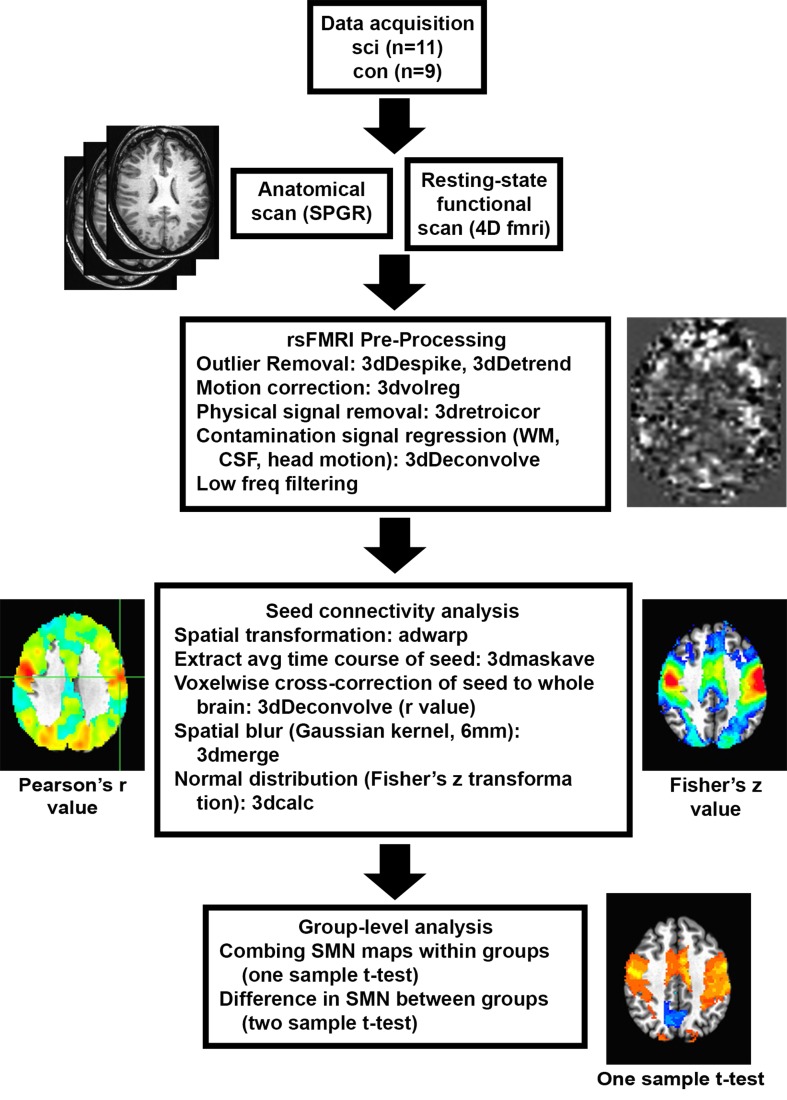
Overview of Data Process. Top to bottom: Data acquisition, preprocessing to obtain individual sensorimotor network maps, and statistical analysis for group-level comparisons. SCI, spinal cord injury; con, control; SPGR, spoiled gradient-recalled echo sequence; fmri, functional magnetic resonance imaging; rsFMRI, resting state functional magnetic resonance imaging; WM, white matter; CSF, cerebrospinal fluid; SMN, sensory motor network.

The preprocessing of MR scanner DICOM data was carried out offline using Analysis of Functional Neuroimaging (AFNI) software (http://afni.nimh.nih.gov.afni) and MATLAB programs (The MathWorks Inc., Natick, MA) as summarized in Fig 1. Preprocessing was initiated by the creation of 3D basic datasets from raw scanner 2D image data files for anatomical (SPGR) and functional data (to3d, AFNI). The parameters required for 3d conversion include number of slices = 41, number of volumes = 240 and repetition time = 2 secs with slices acquired in an interleaved fashion in the z-direction. Signal spike artifacts are then removed from the 3D dataset time series by interpolating data from neighboring time points with spikes defined as data points greater than 4 standard deviations from the mean of the time series (3dDespike, AFNI). The first five volumes of the time series were discarded to accommodate for fluctuations induced while longitudinal magnetization became stabilized.

Subsequent to removal of signal outliers and initial volumes, rigid body correction is performed for head motion along the three translational (x, y, and z) and the three rotational parameters (roll, pitch, and yaw). The motion correction involves default iterated least-square minimization using the higher-order Fourier interpolation with co registration of all functional volumes to base volume 10 of the given datatset (3dvolreg, AFNI). Further, detrending is done to remove mean, linear and quadratic trends to focus the data analysis on signal fluctuation present in the dataset (3dDetrend, AFNI).

The next step is removal of physiological artifacts as they impact detection of brain activation. Independent measurements of cardiac and respiratory variables followed by removal of changes in the image intensity in phase with the physiological cycle is done as part of retrospective image correction (RETROICOR) (3dretroicor, AFNI) [32]. Another source of artifacts is signals from the white matter (WM) and the cerebrospinal fluid (CSF). The removal of WM and CSF signals involves the masks using segmentation done with Statistical Parametric Mapping (SPM) software package for Matlab. The masks created for the WM and the CSF are converted to the resolution of the functional dataset (3dfractionize, AFNI) and the average timecourses within these masks are estimated and regressed out from the rs-fMRI data (3dmaskave and 3dDeconvolve, AFNI). The six-motion vectors were regressed out from each voxel time series (3dDeconvolve, AFNI). Finally, a temporal band-pass filter is applied to allow for low-frequency fluctuations within 0.015–0.1 Hz frequency range and the data was smoothed spatially (FWHM = 6 mm) [33–35].

### Generation of seed-based correlation maps

A seed-based approach was employed to determine the resting-state functional connectivity for selected regions of interest (ROI) [36]. Primary motor and sensory cortical areas were chosen as the ROIs for data analsyis. Time course for each voxel inside any choses ROI is averaged and correlated with all the other voxels in the brain (3dDetrend, AFNI). The seed regions or ROIs were based on Anatomical Automatic Labeling (AAL) template and included, individually, the left and right precentral and postcentral gyrus [37].

The SPGR and rs-fMRI data were spatially transformed to the Talairach template coordinates, (adwarp, AFNI), and resampled to 2-mm isotropic voxels. Correlation maps based on Pearson cross-correlation (r) coefficients were generated. These coefficients were converted to z values via Fisher’s transformation for normal distribution and whole-brain functional connectivity maps were generated for each subject. These individual functional connectivity maps were averaged across both groups of subjects. The functional connectivity maps for every seed were compared at the group level between the SCI patients and healthy volunteers using two-sampled t-tests with p<0.05 considered statistically significant [34]. For determining the cluster size, Monte-Carlo simulation based multiple comparison correction was performed using 3dClustSim program from AFNI (cluster size = 444 voxels).

## Results

**Fig 2:**
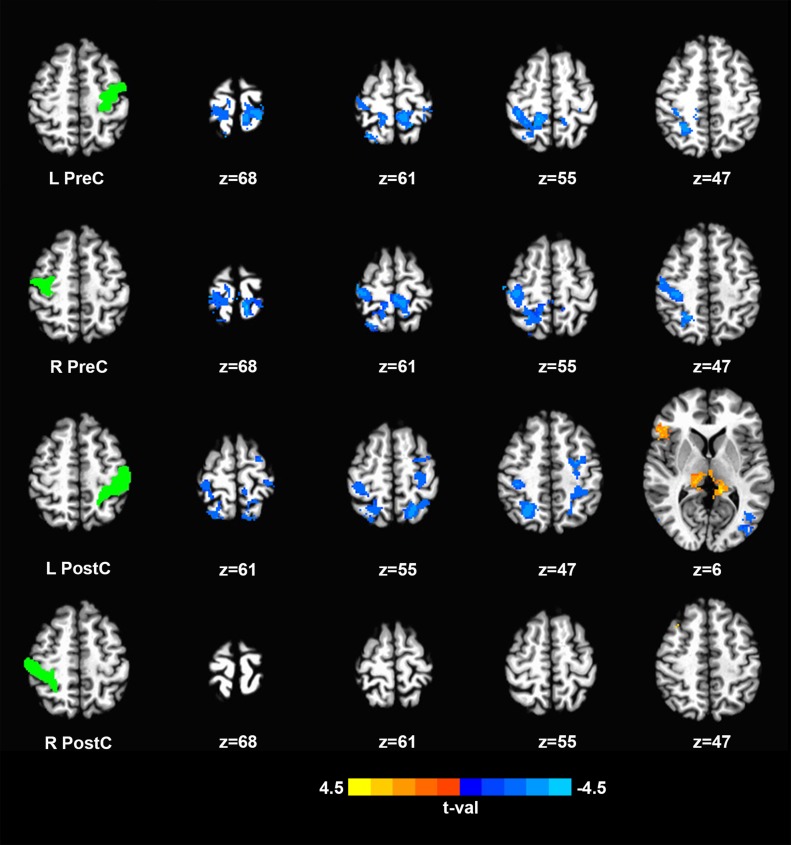
Changes in resting-state functional connectivity of sensorimotor network between SCI patients and controls with each row corresponding to a specific ROI. Left column, Location of ROIs. Remaining columns, Clusters showing significant difference in functional connectivity. Color-coded statistical t-value maps (corrected p<0.05) showing positive and negative correlation (coded in yellow to light blue). Blue colors signifying negative t-values indicating SCI patients have lower connectivity to corresponding ROI compared to controls and vice versa (z-coordinates of cross sections are reported in Talairach space). SCI, spinal cord injury; ROI, region of interest.

In the present study, correlating the average time course for each ROI with all the other brain voxels generated functional connectivity maps for individual subjects. Subsequently, subject-specific correlation maps, generated for each participant, were compared between both groups to determine statistically significant altered connectivity. A two-sampled t-test comparison revealed decreased connectivity in the SCI group across primary sensorimotor areas for both the motor and sensory ROIs as shown in Fig 2 and Table 3.

**Table 3:** Brain regions showing significantly altered functional connectivity in SCI patients.

Seed ROI (gyrus)	SMN Connected Region	Side	Talairach coordinate (RAI)			Cluster size (mm^3^)	Peak *t* value
			*x*	*y*	*z*		
***SCI patients < controls***							
R Precentral	Precentral gyrus	R	-47	16	43	15392	-4.16
	Postcentral gyrus	R	-24	51	48		-4.29
	SMA	R	-10	22	67		-2.31
	Superior parietal lobule	R	-23	53	48		-4.33
	Paracentral lobule	R	-10	36	68		-2.81
	Postcentral gyrus	L	17	37	71		-3.55
	Paracentral lobule	L	10	42	68		-3.91
L Precentral	Precentral gyrus	L	31	25	66	4664	-3.92
	Postcentral gyrus	L	19	35	71		-3.75
	Superior parietal lobule	L	17	49	61		-3.34
	Paracentral lobule	L	9	41	64		-3.38
	Precentral gyrus	R	-35	21	63	6928	-3.43
	Postcentral gyrus	R	-14	41	55		-3.65
	Superior parietal lobule	R	-25	59	62		-4.35
	Paracentral lobule	R	-11	41	57		-3.54
L Postcentral	Precentral gyrus	L	25	21	51	9968	-3.94
	Postcentral gyrus	L	27	39	47		-3.27
	Superior parietal lobule	L	19	57	29		-3.99
	Paracentral lobule	L	8	38	64		-2.71
	Precentral gyrus	R	-35	30	52	6840	-3.35
	Postcentral gyrus	R	-35	30	49		-3.51
	Superior parietal lobule	R	-25	61	60		-4.58
***SCI patients > controls***							
L Postcentral	Thalamus	L	12	31	4	6008	3.50
	Thalamus	R	-13	29	6		3.90

SCI: spinal cord injury; ROI: region of interest; SMN: sensorimotor network; RAI: right-anterior-inferior; SMA: supplementary motor area; p < 0.05, corrected by 3dClustSim.

The motor ROIs included the left and the right precentral gyrus. The left precentral gyrus showed decreased connectivity with bilateral primary motor as well as sensory regions. The same was found to hold true for the right precentral gyrus. Further, for both the motor ROIs, a decrease was noted in the interhemispheric region that serves as a connection between the primary motor and sensory areas.

The sensory ROIs included the left and the right postcentral gyrus. The left postcentral ROI produced similar results when compared with regions of sensorimotor cortex. Similar to motor ROIs, a correlational decrease was noted with the interhemispheric region connecting the primary sensorimotor areas. However, the right postcentral gyrus failed to demonstrate increased or decreased connectivity pattern with other sensorimotor regions. Changing the value to p = 0.1 lead to similar findings in left postcentral gyrus as seen with the other ROIs. For the purpose of our analysis, we have only included results that showed significance at p = 0.05.

In addition, both the left sensory ROI demonstrated altered connectivity with the thalamus. The group comparison showed increased connectivity with thalami on both sides. However, corresponding differences in connectivity with the thalami were not observed for the left or right motor ROIs at the given level of significance (p = 0.5).

## Discussion

The present study has analyzed the sensorimotor network activation patterns during the resting-state in the later stages of SCI. The neural damage in SCI results in structural and functional alterations throughout the neuraxis [6–11, 19]. The present connectivity assessment is based on scans conducted with patients awake yet resting with eyes closed. The data analysis benefits from the absence of a task paradigm during data collection, thereby, removing a potential source of variability [19, 38]. The distinct connectivity patterns observed between the study groups highlights the utility of rs-fMRI in visualizing changes to distant structures of the neuraxis that do not suffer direct damage. The demonstration of altered resting state patterns and the interdependence between task independent (resting) and dependent networks might improve our understanding of the relation between resting state reorganization of cortex and modification of bodily function in patients with SCI [25, 39].

The sensorimotor cortex shows reduced functional connectivity in SCI patients compared to controls in our study. The distortion to input/output signal transmission due to injury might cause spatial shifts of cortical activation areas or reduction in intrinsic activations [40]. The spatial shifts, inherent decreases or a combination of both might play a role in the patterns observed in the present analysis. Evidence from task based functional studies in animals and humans demonstrate the existence of spatial shifts in cortical activation patterns, which tend to gravitate medially and posteriorly. The shifts occur due to expansion of the adjoining innervated parts into deafferented regions of the cortex with expansion of intact areas shown to start as early as days post injury [4, 14, 18, 41, 42]. The detection of modified BOLD signal correlations in the sensorimotor network indicates that the brain undergoes dynamic functional remolding subsequent to distant spinal cord injury.

The presence of altered activation patterns in chronic SCI patients several years post injury shows that neural plasticity leads to lasting alterations to the resting state functional connections between discrete cortical and subcortical structures. The patient group comprises of patients with injury duration of more than two years. The amount of time elapsed might have an influence on the observed activation patterns [43].

Previously, positive correlation has been observed between the severity of spinal cord injury and derangement in measures of structural connectivity like fractional anisotropy (FA) at distant cord regions, indicating severity of the injury influences structural connectivity at distant sites [6]. It is observed that the more severe the spinal cord injury, the greater the distortion in afferent and efferent signal transmission. Our dataset is limited to the complete SCI patients (ASIA A), the most severely injured group. The seed regions used in the analysis are not directly connected to the cord, unlike the structure connectivity example mentioned above. However, we believe that the severity of the injury might have an influence on the resulting change in connectivity patterns observed. Further, all patients had cervical spine injuries, which destroy a greater fraction of the afferent/efferent axons in comparison to injuries to the thoracic cord with subsequent greater effects on the brain.

The ROIs (as per AAL template) chosen to represent the motor cortex included the left and the right precentral gyrus. These ROIs represent primary motor areas. The patient population contained subjects with complete cervical cord injury. This meant that all patients had complete paralysis of lower limbs with varying degrees of function in the upper extremities. It is theorized that the complete lack of inputs and subsequent outputs would manifest as a distinct change on account of the neural plasticity of the human brain and expansion of intact cortical areas to compensate for denervated areas. Hence, measurement of resting correlations of primary motor areas in such a population would yield data with a high probability of measuring significant alterations. The individual precentral gyrus or primary motor area (M1) showed decrease connectivity with their own self, the contralateral precentral gyrus, the inter hemispheric area connecting the two primary motor areas and with the primary sensory areas (S1) on both sides. Our data is supported by demonstration of similar results in acute SCI, pointing to a decline in motor output early in the process. [31]. This might be due to a change in the responsiveness of cortical circuits of inhibitory nature with an increase causing a reduction in the excitation of motor efferents [44].

The ROIs for somatosensory cortex were left and right postcentral gyri, representative of primary sensory cortex (S1). The left postcentral gyrus showed decreased connectivity with primary sensorimotor areas in SCI patients similar to that seen with motor ROIs. There was also a decrease in interhemispheric connectivity between the two primary sensory areas. These findings coupled with the results of both the motor ROIs point to possible decreased interhemispheric communication in the resting state. The decrease is possibly due to loss of synchronization between sensorimotor areas of both the cerebral hemispheres consequent to input/output imbalance. The right postcentral ROI did not show any significant finding at p = 0.05 but showed similar findings at p = 0.1, which could mean that right sensory ROI might be showing the same effects as observed with other ROIs and might benefit from a larger sample size. More importantly, the trend for both sensory ROIs points towards decreased connectivity with sensory and motor areas. Interestingly, the left sensory ROI showed increased connectivity with bilateral thalami. This might be the result of thalami overcompensating for the decrease in input received from caudal structures and could be influenced by factors such as disinhibition of latent synapses or sprouting of new synapses. Furthermore, many SCI patients complain of neuropathic pain, which might have a resultant effect on functional connectivity at rest [45].

A number of factors warrant consideration in furthering our understanding of the neural plasticity in chronic SCI. The present study is constrained by the relatively few number of subjects in both groups. The small sample size raises the issue of statistical power for which power analysis using the bootstrap method was performed (S1 File). The result showed that approximately 10 subjects per group are required to achieve a power of 0.80 for detection of group difference (for detailed explanation of power analysis refer to S1 File). The sample size in this study is roughly equivalent to the number calculated by power analysis. However, the study could benefit form having a larger sample size. Further, the severity and the level of injury could impact the progression of connectivity alterations at supra spinal levels post SCI [6]. The present analysis was limited to cervical cases but comparing a larger dataset of SCI patients across different levels with varying ASIA grades would help to better understand the effect of these variables. The time since injury also has implications in the reorganization of activation maps [43]. This study did not account for neuropathic pain experienced by SCI patients. Since, pain pathways involve thalamus as an important conduit, subsequent resting data analysis might need to explore this phenomena [45]. This study solely looks at the plasticity of the brain without analyzing the reorganization of the spinal cord post SCI. The functional changes in cord might have a role in play in the resultant lasting reorganization in chronic patients. Also, mood alterations have been known to affect cortical connectivity maps and might be an influence in SCI patient cohort. Lastly, we have not included handedness data in the present study. We recognized that handedness could potentially influence resting state sensorimotor connectivity and feel this data would be valuable in additional studies moving forward. In continuation of our work with SCI patients, some of the above mentioned concerns would be included to improve upon the understanding of the cortical reorganization and the underlying pathophysiologic mechanism behind observed activation changes.

## Conclusion

The current study has compared the resting-state functional data between chronic cervical SCI patients and healthy controls. We show alterations in spontaneous cortical activation patterns suggesting a possible SCI-induced reorganization on account of neural plasticity. The demonstration of the cortical response to SCI highlights the potential of rs-fMRI for studying neuroplasticity in the brain subsequent to injuries to distal elements of the central nervous system.

## Supporting Information

S1 File Power Analysis. (PDF)
