# Supplementary material for: Alterations in Cortical Sensorimotor Connectivity following Complete Cervical Spinal Cord Injury: A Prospective Resting-State fMRI Study
Source: PLoS One. 2016 Mar 8;11(3):e0150351. doi: 10.1371/journal.pone.0150351 (PMC4783046; doi:10.1371/journal.pone.0150351)

Power Analysis

1.1 To conduct this power analysis, we used the bootstrap power analysis method. It involved extensive use of previously collected datasets, which included 11 spinal cord injury subjects (SCI) and 9 healthy control subjects (HC). For each subject, rs-fcMRI data was collected and pre-processed. For this study, there were three specific ROI's of interest: the right precentral gyrus, the left precentral gyrus, and the left postcentral gyrus. The functional connectivity (FC) for each of these 3 ROI's was calculated for each subject and used as input to the bootstrap resampling procedure (see flowchart below) to estimate statistical power. Note: all FC values were Fisher z-transformed to be approximately normally distributed, as required by the statistical analyses. Here, to correct for multiple inferences, we used the Bonferroni correction to set the null hypothesis rejection probability threshold at $pThr = \alpha / NROI = 0.05 / 3 = 0.0167$ (pThr = p threshold; NROI = number of ROIs used).

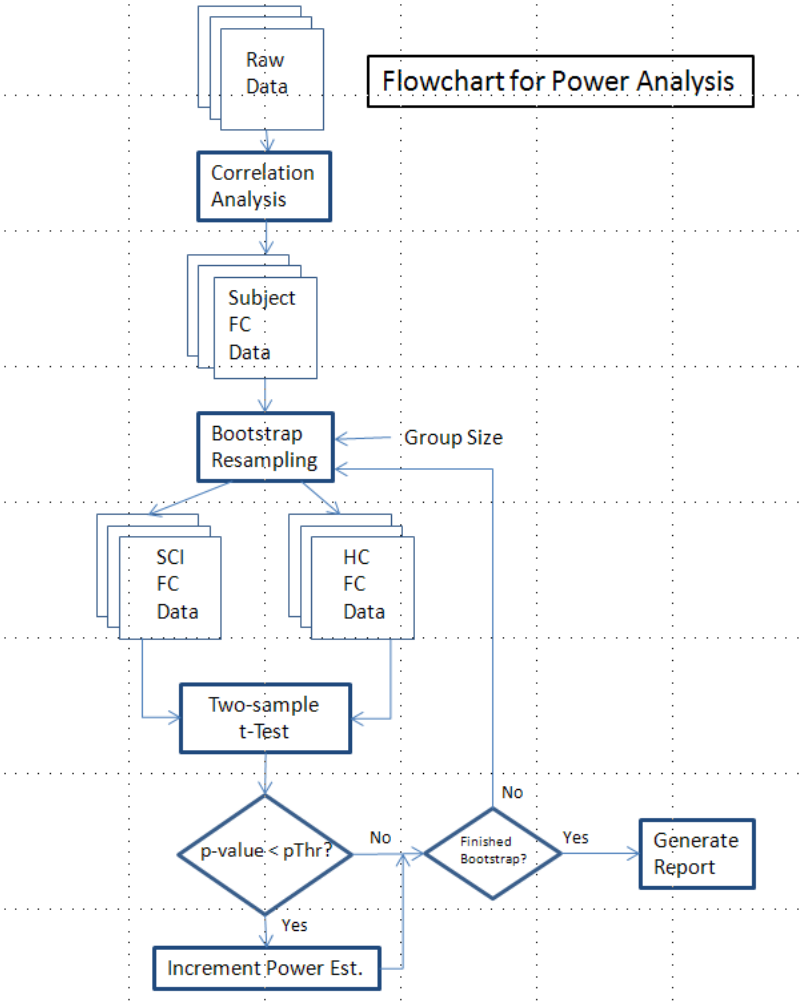

1.2 For each ROI, and for each hypothetical group size, NBOOT = 1000 bootstrap iterations were performed, where the SCI and HC cohorts were selected at random, and with replacement, from the original pools of 11 and 9 subjects, each (NBOOT = number of iterations). A two-sample t-test was performed for comparing the FC data from the SCI and HC cohorts, for each ROI. The null hypothesis was rejected when the t-test p-value was less than pThr. For each bootstrap iteration, we recorded whether the null hypothesis was rejected at significance level pThr, in which case the estimated power was incremented by 1/NBOOT. This procedure was repeated for different hypothetical group sizes, and for each ROI. The results are plotted in the figure below, which shows the power to detect a difference between the SCI and HC groups due to differences in FC. Different ROI's are represented by different colors. Note that approximately 10 subjects per group are required to achieve a power of 0.80 for detection of group difference.

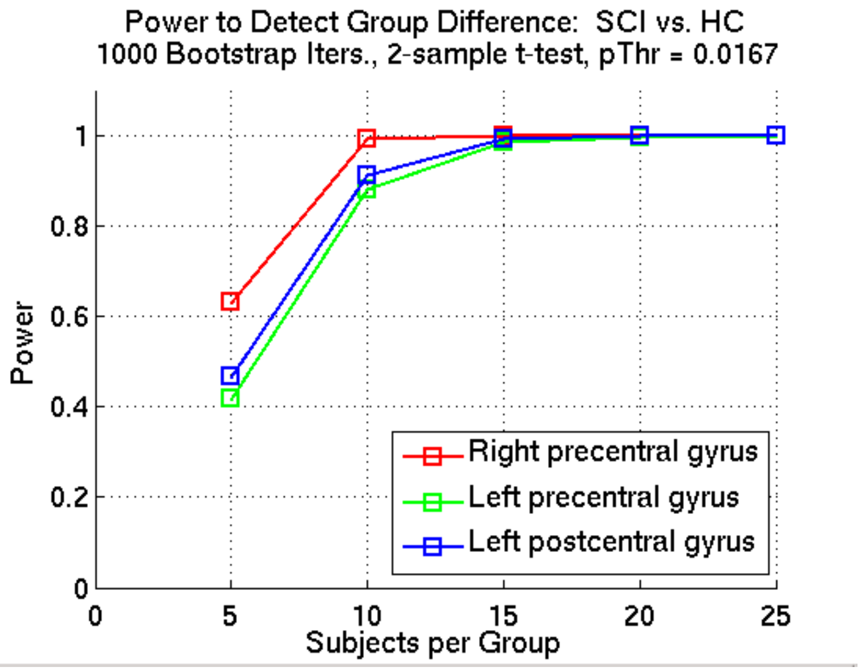

Supplement: S1 File — (PDF) [file pone.0150351.s001.pdf]
